# Unweaving the population structure and genetic diversity of Canadian shrub willow

**DOI:** 10.1038/s41598-022-20498-9

**Published:** 2022-10-14

**Authors:** Emily K. Murphy, Eduardo P. Cappa, Raju Y. Soolanayakanahally, Yousry A. El-Kassaby, Isobel A. P. Parkin, William R. Schroeder, Shawn D. Mansfield

**Affiliations:** 1 Department of Wood Science, Faculty of Forestry, University of British Columbia, Vancouver, BC Canada; 2 Instituto Nacional de Tecnología Agropecuaria, Instituto de Recursos Biológicos, Centro de Investigación en Recursos Naturales, Buenos Aires, Argentina; 3 Consejo Nacional de Investigaciones Científicas y Técnicas, Buenos Aires, Argentina; 4 Department of Forest and Conservation Sciences, Faculty of Forestry, University of British Columbia, Vancouver, BC Canada; 5 Indian Head Research Farm, Agriculture and Agri-Food Canada, Indian Head, SK Canada; 6 Saskatoon Research and Development Centre, Agriculture and Agri-Food Canada, Saskatoon, SK Canada; 7 Department of Botany, University of British Columbia, Vancouver, BC Canada

**Keywords:** Plant biotechnology, Sequencing, Plant biotechnology, Plant evolution, Biotechnology, Plant sciences

## Abstract

Perennial shrub willow are increasingly being promoted in short-rotation coppice systems as biomass feedstocks, for phytoremediation applications, and for the diverse ecosystem services that can accrue. This renewed interest has led to widespread willow cultivation, particularly of non-native varieties. However, Canadian willow species have not been widely adopted and their inherent diversity has not yet been thoroughly investigated. In this study, 324 genotypes of *Salix famelica* and *Salix eriocephala* collected from 33 sites of origin were analyzed using 26,016 single nucleotide polymorphisms to reveal patterns of population structure and genetic diversity. Analyses by Bayesian methods and principal component analysis detected five main clusters that appeared to be largely shaped by geoclimatic variables including mean annual precipitation and the number of frost-free days. The overall observed (*H*_*O*_) and expected (*H*_*E*_) heterozygosity were 0.126 and 0.179, respectively. An analysis of molecular variance revealed that the highest genetic variation occurred within genotypes (69%), while 8% of the variation existed among clusters and 23% between genotypes within clusters. These findings provide new insights into the extent of genetic variation that exists within native shrub willow species which could be leveraged in pan-Canadian willow breeding programs.

## Introduction

Shrub willow (*Salix* spp.) occur naturally in mesic areas adjacent to watercourses and wetlands across much of North America^1^. They were traditionally used in the production of woven baskets and as a source for analgesics^2,3^. Willow has been planted extensively as riparian buffers and shelterbelts in agricultural regions, and it has been promoted as a promising biomass feedstock for bioenergy applications^4^. The carbon dioxide that is released from burning willow wood is largely offset by its fixation during photosynthesis, making willow biomass a nearly carbon–neutral energy source^5^. In addition, shrub willow responds well to coppicing and is capable of reaching maximum annual growth rates at high planting densities in just a few growing seasons^6^. However, the non-native species that are routinely deployed can suffer from pests and disease, and may elicit concerns with regards to invasiveness^7^. As locally adapted genotypes offer unparalleled resistance to climatic and biotic stressors, the development of genetic resources for native willow species could facilitate the development of more resilient and productive commercial willow cultivars adapted for Canadian landscapes^8^.

Willow are dioecious and obligate outcrossers. There are approximately 450 recognized willow species in the world, falling into six sub-genera, of which over 100 occur in North America^9^. Canada alone has 76 native species^10^. Despite this enormous natural diversity, native willow taxa have received comparatively little research attention and few studies have sought to exploit this extensive variation. Three related shrub willow species occur across much of the temperate and boreal regions of North America: *Salix eriocephala* Michx. (2*n* = 2 × = 38; subgenus *Vetrix*; section *Cordatae*), *Salix famelica* (C.R. Ball) Argus (2*n* = 2 × = 38; subgenus *Vetrix*; section *Cordatae*), and *Salix prolixa* Andersson (2*n* = 2 × = 38; subgenus *Vetrix*; section *Cordatae*)^11^. Previously, these taxa have been circumscribed as subspecies: *S. eriocephala* ssp. *eriocephala* (mainly in Ontario, Quebec, New Brunswick, Nova Scotia, Prince Edward Island, and Newfoundland and Labrador), *S. eriocephala* ssp. *famelica* (Alberta, Saskatchewan, Manitoba, and Ontario), and *S. eriocephala* ssp. *mackenzieana* (Hooker) Dorn (Alaska, Yukon, British Columbia, and the Northwest Territories). However, the most recent taxonomic revision of North American *Salix*^12^ recognizes these as three separate species: *S. eriocephala *sensu stricto, *S. famelica*, and *S. prolixa*. Argus et al*.*^12^ note that although a small portion of the ranges of these taxa overlap, the distribution and morphology are sufficiently distinct to support separate species designations; however, genetic support for this has been lacking^13^.

The largest subgenus, *Vetrix*, are the most diverse and currently has an estimated 125 species. Only a small fraction of these is currently employed in short-rotation coppice (SRC) systems, whereby willow productivity is maximized by exploiting the propensity of shrub willow to regrow quickly after cutting the stems near the base. Over the past fifty years, willow breeding efforts in New Zealand, Sweden, the UK, and the USA have resulted in the development and deployment of both intra- and interspecific hybrids with important agronomic traits needed for SRC^8,14–16^. Yields of 4–6 oven dry tonnes (odt) ha^−1^ y^−1^ have been reported for hybrids when grown in commercial biomass production systems, while experimental plots have produced as much as 24–30 odt ha^−1^ y^−1^ in some studies^17^. On Prince Edward Island, the willow cultivars *S. viminalis* (5027) and *S. dasyclados* (SV1) can yield up to 18–20 odt ha^−1^ y^−1^ when planted as riparian buffer strips along watercourses^18^. Consequently, shrub willow are increasingly garnering attention in SRC systems for their uses in managing wetlands^19^, intercepting excess nutrient runoff^20^, remediating industrial soils and wastewater^21,22^, and improving the biodiversity of birds and insects^23^.

Despite the economic and ecological importance of willow, the development of genetic resources has lagged behind other woody species such as poplar and eucalyptus^24^. Nonetheless, molecular markers have been used to explore the genetic diversity in willow species, including those in a study of European *S. viminalis* that formed the foundations for the development of an association mapping population^25^. As in other tree species with well-developed genetic resources, single nucleotide polymorphisms (SNPs) have become the preferred marker as they are both easily distinguished and abundant across the genome, providing broad coverage of the species’ genetic architecture by capturing both adaptive and neutral diversity. For example, SNP markers were recently used to assess the genetic diversity of *S. purpurea* from Europe that has naturalized in the northeastern USA^26^. Genome-wide approaches are advantageous because they can simultaneously discern population structure and facilitate marker development for molecular breeding.

At Agriculture and Agri-Food Canada (AAFC), in-house willow resources are being managed to develop biomass feedstocks for bioenergy and environmental applications, which has led to the establishment of the Agriculture Canada *Salix* (AgCan*Salix*) germplasm collection. The priority has been to amass large genetically diverse base populations from which parents can be selected to develop locally adapted genotypes tailored for specific applications. Historically, progeny selection has been performed both under controlled and field environments for traits such as cold hardiness, salinity tolerance, and disease resistance. Moving forward, the objectives are to expand the breeding pool and select new cultivars with agronomic traits that maintain high biomass yields.

Willow breeding programs have already had some success in developing new cultivars optimized for SRC production. For example, the Swedish breeding pipeline has generated several high-yielding willow varieties with improved disease resistance and better frost tolerance by crossing genotypes from across Europe, most notably from central and eastern Russia^27^. Similar success has been achieved at Rothamsted in the UK and in upstate New York in the USA, where commercial cultivars have been established that perform well in those respective climates^24,28^. As such, there is every reason to believe that concerted efforts with Canadian shrub willow germplasm will help cultivate a productive short-rotation coppice industry in Canada.

Given that the AgCan*Salix* collection exhibits vast phenotypic diversity in terms of growth, phenology, physiology, and wood chemistry^4,29,30^, we anticipated that there would be considerable underlying genetic diversity to enable future breeding work. In this study, we investigated the geographic pattern of genetic diversity of *S. famelica* and *S. eriocephala* using genotyping-by-sequencing (GBS) to: (1) determine whether geoclimatic variables influenced the population structure, and (2) determine the level of genetic variation within and among populations. By uncovering the extent of diversity within the AgCan*Salix* collection and the relatedness of genotypes, this work will bolster future efforts to breed willow varieties with superior adaptive traits for a host of Canadian climatic regions. The ultimate aim is to develop multi-functional feedstocks that can simultaneously support a burgeoning bioenergy industry and also deliver environmental benefits.

## Materials and methods

### AgCan*Salix* collection

AAFC assembled a collection of Canada’s native willow germplasm from wild populations of *S. amygdaloides*, *S. bebbiana*, *S. discolor*, *S. eriocephala*, *S. famelica*, *S. interior*, and *S. petiolaris* during the summer of 2012. This collection, known as AgCan*Salix*, includes willow genotypes from Alberta, Saskatchewan, Manitoba, Ontario, Quebec, New Brunswick, Prince Edward Island, Nova Scotia, and Newfoundland and Labrador (Table S1; Fig. S1).

For *S. famelica* and *S. eriocephala* collectively, 34 sites were sampled with 15 genotypes per site of origin for a total of 510 genotypes representing the range across Canada. The geographic range of this collection spanned 10° in latitude (45–55 °N), 55° in longitude (57–113 °W), and 796 m in elevation (4–800 m). Sampling was done across a west–east transect to capture the geographic variation that these species occupy. The risk of clonal sampling was mitigated by selecting genotypes that were separated by a minimum of 1 km. The cuttings were collected during the dormancy period, bagged separately, and stored at − 4 °C. In the spring, dormant cuttings were dipped in rooting hormone powder (0.8% indole-3-butyric acid, Plant Products Co. Ltd., Brampton, Ontario, Canada) and induced to root in Spencer-Lemaire rootrainers (Beaver Plastics, Acheson, Alberta, Canada). Willow plants were grown in a greenhouse located at Indian Head, Saskatchewan (51 °N, 104 °W; elevation 605 m) with daytime and nighttime temperatures maintained at 23 and 18 °C, respectively, and a relative humidity of 40%. After two months of growth in the greenhouse, the willow plants were transferred to a shade house and allowed to undergo natural senescence. In late October, the frozen root plugs were individually bagged and stored at − 4 °C until the following spring when a common garden plot was established at Indian Head.

### Geoclimatic data at sites of origin

Long-term climate normals (1981–2010) were obtained from nearby government-run weather stations operated by Environment Canada^31^ to provide environmental variables associated with the sites of origin, including frost-free days (FFDs, number of days), mean annual precipitation (MAP, mm), mean summer temperature (MST, °C), and growing degree days > 5 °C (GDD, °C) (Table S1). We define frost-free days as the number of days with a minimum temperature above 0 °C, a proxy for growing season length. The MAP range for the sites of origin varied markedly from 316 to 1709 mm. The MST ranged from 9.4 to 15.5 °C, values of FFD varied from 151 to 210 days, and the GDD fluctuated from 1023 to 1955 °C.

### DNA extraction and genotyping-by-sequencing (GBS)

For this study on the genetic diversity of the AgCan*Salix* collection, 324 genotypes were selected (8–10 genotypes per site of origin, from 33 sites of origin). Young leaf tissues from the common garden were collected in Eppendorf tubes, immediately flash-frozen in liquid nitrogen, and stored at − 80 °C.

DNA was isolated from frozen leaf tissue using the CTAB method^32^ and quantified using a PicoGreen dsDNA kit (Molecular Probes, Life Technologies Inc., Burlington, Ontario, Canada). Library generation was based on the 96-plex GBS protocol described by Poland et al*.*^33^, employing a double-digest with *PstI* and *MspI* as the restriction enzymes. The resulting libraries were then sequenced using an Illumina HiSeq 2000 system (Illumina Inc., San Diego, California, USA).

Putative GBS markers were identified following the TASSEL 5.0 GBSv2 Discovery Pipeline^34^, using the *Salix purpurea* v1.0 genome^35^ as a mapping reference. This resulted in an initial dataset comprising 55,453 putative markers. Quality filtering and missing marker imputation were completed using the *synbreed* package^36^ in R version 3.5.1^37^. First, markers were recoded to reflect the number of copies of a reference allele: aa = 0, Aa = 1, and AA = 2. Then, markers with a minor allele frequency (MAF) below 1% and > 50% missing data were removed. Missing markers (16.2%) were imputed using the random method in the *codeGeno* function, in which the missing values were sampled from the existing marginal allele distribution. This resulted in a final filtered set of 26,016 polymorphic markers for 324 genotypes. Among these markers, the highest number of genotypes with missing data was 162 out of 324 total genotypes. However, most of the markers had far fewer genotypes requiring imputation. The mean number of markers with imputed data was 52 out of 324 genotypes, with a standard deviation of 49.

### Data analysis

To gain preliminary insights into population structure, a principal component analysis (PCA) was carried out using the *glPca* function in the R package *adegenet*^38^. Genotypes were broadly classified as either western or eastern, based on the reported ranges for *S. famelica* and *S. eriocephala* discerned by examining plant morphology^39^.

The population structure was assessed using a Bayesian approach for posterior inference implemented in fastSTRUCTURE^40^. The default convergence threshold was selected and runs were performed for a number of clusters ranging from *K* = 1 to *K* = 10 using all 324 genotypes. The output was then visualised using *Distruct for many K’s* as implemented in CLUMPAK^41^. The optimal value of *K* was determined using the *chooseK.py* algorithm, from which *K* = 4 was identified as the number of model components that maximized marginal likelihood, and *K* = 5 was the number of model components used to explain structure in data. The R package *ggplot2*^42^ was then used to plot the average membership across fastSTRUCTURE runs at *K* = 5.

Pie charts representing admixture results for K = 5 were generated by the R package scatterpie^43^ and were plotted onto a geographical map of Canada imported from the R package rnaturalearth^44^ using R packages ggplot2^42^ and ggspatial^45^. The species ranges proposed by Argus^39^ were added manually onto this map using Adobe Illustrator (Adobe Inc., San Jose, CA, USA).

For comparison, another evaluation of the population structure was carried out by performing a discriminant analysis of principal components (DAPC) using the *dapc* function in the R package *adegenet*^38^. Here, the optimal number of clusters, *K*, was determined for the 324 genotypes sourced from 33 sites of origin using the *find.clusters* function in the R package *adegenet*^38^. *K* = 5 was inferred by applying the Elbow method, a heuristic commonly used to manually evaluate the number of clusters as the point of inflection on a plot of the Bayesian information criterion (BIC) values against sequential numbers of clusters (*K* values).

To evaluate genetic diversity, the allelic frequency (p and q), MAF, expected heterozygosity (*H*_*E*_), observed heterozygosity (*H*_*O*_), Nei’s gene diversity (GD), polymorphism informative content (PIC), and the χ^2^ statistic for the Hardy–Weinberg equilibrium test and its corresponding *P*-value were calculated for each cluster using the R package *snpReady*^46^. To determine the level of population genetic differentiation, pairwise fixation indexes (*F*_*ST*_) for each cluster were calculated based on the method of Weir and Cockerham^47^, using the R package *StAMPP*^48^.

The correlation between genetic distance (pairwise *F*_*ST*_ values) and geographic distance among 33 sites of origin was assessed by performing an isolation-by-distance (IBD) analysis using the R package *adegenet*^49^. The significance of the associations was tested based on a Mantel test with 10,000 permutations using the *mantel.randtest* function. Local density was plotted with two-dimensional kernel density estimations determined using the R package *MASS*^50^.

To calculate the extent of hierarchical population structure, an analysis of molecular variance (AMOVA) was performed based on the infinite alleles model (*F*-statistics) using the R package *poppr*^51^. The AMOVA partitioned variation among geographic clusters, between genotypes within clusters, and within genotypes.

To examine the influence of climate at the sites of origin on genetic structure, a redundancy analysis (RDA) was conducted using the R package *vegan*^52^ following the methodology described by Forester et al*.*^53^ and using the cluster assignments from the Bayesian analysis.

Plots were formatted using Adobe Illustrator (Adobe Inc., San Jose, CA, USA).

## Results

### Population structure

**Figure 1:**
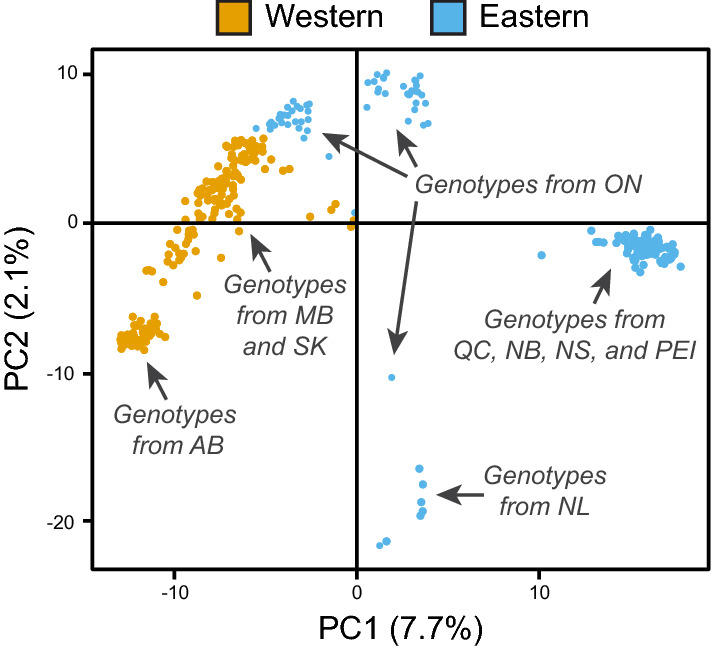
Principal component analysis (PCA) showing 324 genotypes of *S. famelica* and *S. eriocephala*, plotted as western and eastern groups, separated along the first two principal component (PC) axes (explaining 7.7% and 2.1% of the total variation, respectively). AB, Alberta; SK, Saskatchewan; MB, Manitoba; ON, Ontario; QC, Quebec; NB, New Brunswick; NS, Nova Scotia; PEI, Prince Edward Island; NL, Newfoundland and Labrador.

From the set of 324 *S. famelica* and *S. eriocephala* genotypes, 26,016 polymorphic markers were obtained that were distributed evenly across all 19 chromosomes. Using this dataset, we set out to characterize the AgCan*Salix* collection by examining the population structure with PCA (Fig. 1). Genotypes were assigned as either western or eastern based on the reported ranges determined previously from plant morphology^39^. The first two factors from this analysis explained 9.8% of the total variation: PC1, which explained 7.7% of the variation and corresponded to a longitudinal divide, and PC2, which explained 2.1% of the variation and reflected more complex contributions of biogeography. Many of the eastern genotypes formed a compact cluster, although some occurred separately, including those from Newfoundland and Labrador. The majority of western genotypes grouped loosely, while those from Alberta formed a compact cluster. Some of the genotypes from Ontario, which were initially assigned to the eastern group, clustered with western genotypes while others were more scattered, indicating that Ontario genotypes may represent a transition between the western and eastern groups.

**Figure 2:**
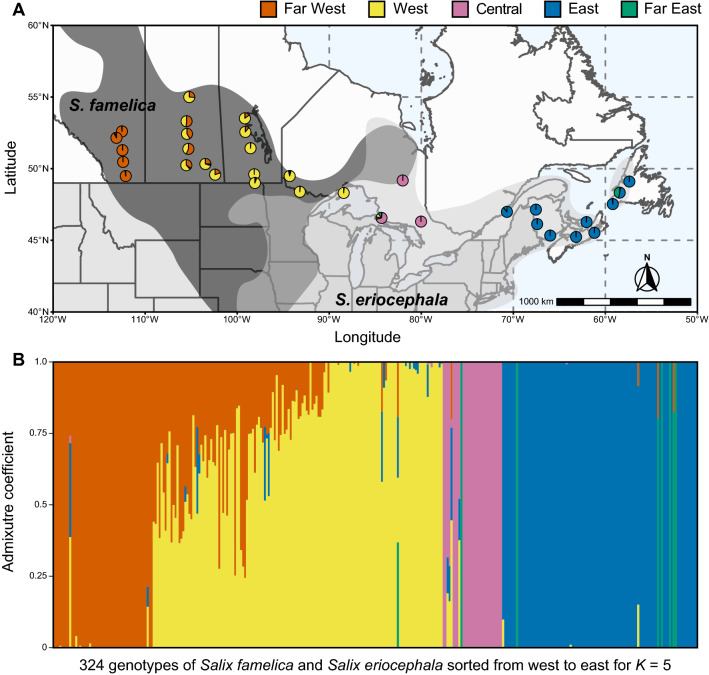
Inference of population structure based on 26,016 polymorphisms in 324 genotypes of *S. famelica* and *S. eriocephala* from 33 sites of origin using Bayesian simulations. (**A**) Pies show mean admixture results (*K* = 5) across genotypes for each site of origin. Shaded areas indicate the species distributions reported in Argus^39^. Map generated using R packages ggplot2^42^, scatterpie^43^, rnaturalearth^44^, and ggspatial^45^. (**B**) Distribution of fastSTRUCTURE-defined clusters among 324 genotypes, sorted from west to east, for *K* = 5 where the y-axis indicates the estimated admixture coefficients for each genotype.

Next, we used fastSTRUCTURE to further assess the population structure and evaluate genetic variation within the collection. This analysis revealed the existence of five clusters, based on the optimal model complexity (Fig. 2A). The proportion of each individual genotype’s Bayesian assignment contributing to each cluster (*K* = 5) is depicted in Fig. 2B. As these largely corresponded to geographic areas, we assigned the following cluster names: Far West (orange), West (yellow), Central (pink), East (blue), and Far East (green). Far West included 68 genotypes from Alberta; West included 128 genotypes from sites in Saskatchewan, Manitoba, and northwestern Ontario; Central included 28 genotypes from central Ontario; East included 92 genotypes from sites in Quebec, New Brunswick, Nova Scotia, Prince Edward Island, and Newfoundland and Labrador; and the final cluster, Far East, had two genotypes from Ontario, one from Quebec, and five from Newfoundland and Labrador. For comparison, the results of fastSTRUCTURE runs for *K* = 2 to *K* = 10 are provided as Fig. S2. Overall, the genotypes from Ontario and Newfoundland and Labrador displayed the greatest admixture, while those from Alberta, New Brunswick, and Nova Scotia had the least admixture.

**Figure 3:**
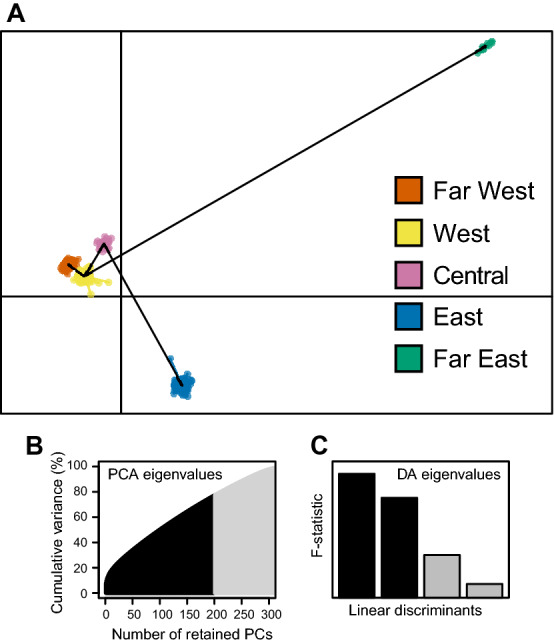
(**A**) Discriminant analysis of principal components (DAPC) scatterplot showing positions of 324 *S. famelica* and *S. eriocephala* genotypes along the first two linear discriminants for *K* = 5. Each dot represents a genotype and a minimum spanning tree (black line) based on the squared distances between the inferred geographic clusters. (**B**) Principal component analysis (PCA) eigenvalues plot shows that 77.3% of the cumulative variance can be explained by the first 200 retained principal components (PCs). (**C**) Discriminant analysis eigenvalues plot shows the proportion of total variance captured by the first two linear discriminants.

As a further confirmation of the population structure, a discriminant analysis of principal components (DAPC) was performed (Fig. 3A). This analysis was conducted by retaining the first 200 principal components with the first two linear discriminants accounting for 77.3% of the cumulative variance (Fig. 3B, C). In agreement with the Bayesian analysis, this method identified the optimal number of clusters to be *K* = 5 (Fig. S3). Again these clusters were reflective of geography such that the same five names were assigned: Far West, West, Central, East, and Far East. The membership of these groups was identical to those in the Bayesian analysis except that five genotypes from Alberta fell into the West cluster rather than Far West. As was observed with the PCA analysis, the western genotypes grouped together, apart from the eastern genotypes. Strikingly, this analysis showed that the Far East cluster was by far the most divergent of all the clusters, based on the minimum-spanning tree which represents the between-group differentiation.

### Genetic diversity

Substantial levels of genetic polymorphism were observed among the collection of *S. famelica* and *S. eriocephala* genotypes (Table 1). For the five clusters, the expected heterozygosity ranged from 0.161 to 0.189, while the observed heterozygosity ranged from 0.125 to 0.162. Nei’s gene diversity (GD) and mean PIC were 0.179 and 0.150 for the collection, respectively, while the MAF ranged from 0.112 to 0.133 with an overall MAF of 0.121 across the entire collection. The conditions of Hardy–Weinberg equilibrium were not met for all five clusters.

**Table 1:** Population genetic diversity summary statistics for 324 *S. famelica* and *S. eriocephala* genotypes within the five geographic clusters identified by Bayesian analysis.

Cluster	*N*	p	q	MAF	*H*_*E*_	*H*_*O*_	GD	PIC	χ^2^
Far West	68	0.121	0.879	0.115	0.167	0.125	0.167	0.140	7.523 (0.300)
West	128	0.115	0.885	0.113	0.167	0.125	0.167	0.140	14.306 (0.263)
Central	28	0.120	0.880	0.112	0.161	0.130	0.161	0.132	2.514 (0.294)
East	92	0.127	0.873	0.115	0.165	0.125	0.165	0.138	9.481 (0.292)
Far East	8	0.146	0.854	0.133	0.189	0.162	0.189	0.153	0.945 (0.309)
Overall	324	0.121	0.879	0.121	0.179	0.126	0.179	0.150	41.322 (0.109)

*N* is the total number of genotypes per site; allelic frequency (p and q). MAF, minor allele frequency; HE, expected heterozygosity; HO, observed heterozygosity; GD, Nei’s gene diversity; PIC, polymorphism informative content. χ^2^ statistic for the Hardy–Weinberg equilibrium (HWE) test and the associated *P*-value, shown in brackets.

Next, we performed pairwise *F*_*ST*_ comparisons between the five clusters, with the lowest *F*_*ST*_ observed between Far East and East (− 0.049, Table 2). The highest *F*_*ST*_ was observed between East and Central (0.097), followed closely by West and Central (0.090). This could again be an indication that the Central genotypes represent a transition zone from west to east. Alternatively, the genetic diversity across this region could be underestimated due to the relatively low number of genotypes and sites of origin included from Ontario and southern Quebec. Significant isolation-by-distance was evident across the range, and the correlation between geographic distance and *F*_*ST*_ was high (*r* = 0.887, *p* = 0.001, Fig. S4).

**Table 2:** Pairwise *F*_*ST*_ values among five geographic clusters in Canada for *S. famelica* and *S. eriocephala* identified by Bayesian analysis.

	Far West	West	Central	East
**West**	0.064			
**Central**	0.079	0.090		
**East**	0.064	0.069	0.097	
**Far East**	− 0.006	− 0.035	− 0.003	− 0.049

The hierarchical AMOVA revealed that 8.2% of the genetic variance existed among the five clusters identified by the Bayesian analysis (Table 3). Greater proportions of genetic polymorphisms were observed between the clusters (22.9%) and within genotypes (68.9%). As further support for these conclusions, the highest phi-statistic values resulted for the variance between clusters and within genotypes. However, as a caveat, only the analysis of variance within genotypes was associated with a significant *p*-value (< 0.01).

**Table 3:** Hierarchical analysis of molecular variance (AMOVA) between five geographic clusters of *S. famelica* and *S. eriocephala* identified by Bayesian analysis*.*

Source of variation	Degrees of freedom	Sum of squares	Mean squares	Variance components	% Variance	Phi statistic Φ_*ST*_	*P-*value^†^
Among clusters	4	100,797.4	25,119.31	195.0	8.2	0.08	> 0.01
Between genotypes within clusters	319	871,394.3	2731.6	544.8	22.9	0.25	> 0.01
Within genotypes	324	532,027.5	1642.1	1642.1	68.9	0.31	< 0.01
Total	647	1,504,219.2	2324.9	2381.9	100		

^†^Significance tests were performed with 999 permutations.

### Influence of geoclimatic variables

**Figure 4:**
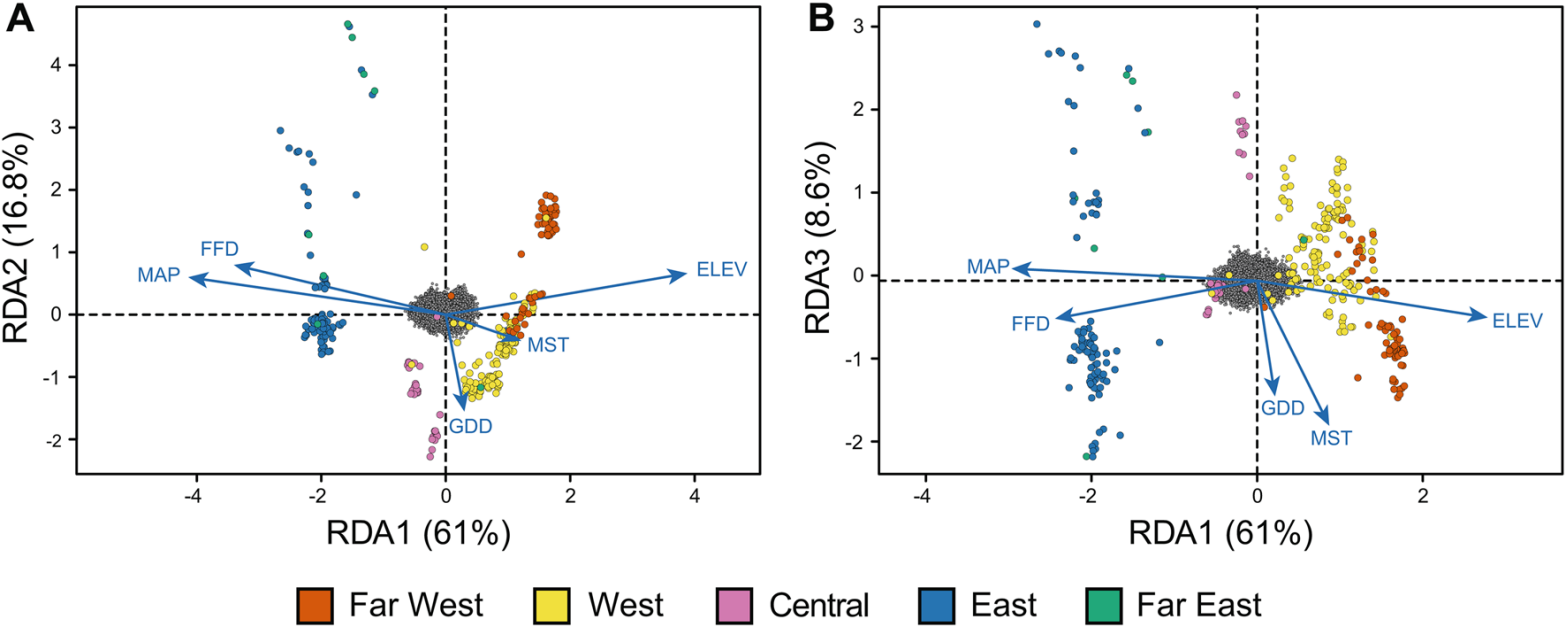
Redundancy analysis (RDA) ordination plots showing the relationship between population structure and environmental variables at the sites of origin along (**A**) RDA axes 1 and 2 (explaining 77.8% of the total variation), and (**B**) axes 1 and 3 (explaining 69.6% of the total variation). The dark grey cluster at the centre of each plot represents the 26,016 polymorphisms, while each dot represents a genotype (324 in total)*,* coloured by geographic cluster as shown in the legend. Blue arrows represent five environmental variables: mean annual precipitation (MAP), frost-free days (FFD), growing degree days (GDD), mean summer temperature (MST), elevation (ELEV).

RDA analysis was performed in an effort to tease apart the impact of geoclimatic variables on population structure. The first three constrained axes explained 86.4% of the total variance. RDA1 (x-axis, Fig. 4A, B) accounted for 61% of the variation and corresponded to a west–east divide of clusters. Genotypes from Central, East, and Far East were positively correlated to mean annual precipitation (MAP) and frost-free days (FFD). By contrast, genotypes from the West and Far West clusters were sourced from higher elevations (ELEV) and were characterized by higher mean summer temperatures (MST). RDA2 (y-axis, Fig. 4A) explained 16.8% of the variation and revealed a positive relationship for Central genotypes with growing degree days (GDD). RDA3 (y-axis, Fig. 4B) revealed considerable separation within each cluster, particularly for the East and the Far East clusters, that was largely correlated with GDD and MST. Overall, this analysis showed that MAP, FFD, and ELEV were strongly related to the population structure and genetic diversity observed among the *S. famelica* and *S. eriocephala* genotypes.

## Discussion

### Distribution of Canadian shrub willow

To our knowledge, this is the most comprehensive range-wide genetic study of *S. famelica* and *S. eriocephala* to date. This study examined 324 willow genotypes sourced from an expansive geographic range (~ 4000 km) spanning much of the temperate and boreal zones of Canada. Our results reveal that the AgCan*Salix* collection falls into a clear pattern of western and eastern genotypes and simultaneously provides genetic evidence to support separate designations for *S. famelica* and *S. eriocephala*.

The contemporary distribution of these taxa has been shaped by both ongoing and historical processes. As the majority of the present-day range of these species was covered by the Laurentide Ice Sheet during the Pleistocene epoch^54^, the Canadian genotypes likely arose following northward expansion from glacial refugia. Notably, Allard and Leonard^55^ described disjunct *S. eriocephala* populations as far south as Georgia and Alabama in the USA. Following the northward range expansion, there may have been physical and/or climatic barriers that contributed to genetic isolation and divergence. Furthermore, it is likely that willow habitats have been drastically altered by centuries of human colonization and extensive changes to the landscapes across North America^56^.

In this study, willow genotypes sourced from broad natural ranges were examined using Bayesian and principal component analyses to elucidate population structure. Both approaches captured five clusters corresponding to five geographic areas. At present, the AgCan*Salix* collection does not include genotypes from west or north of the Rocky Mountains. Further work in this region is needed to examine whether any additional western cluster(s) may exist or whether these genotypes would group with those from Alberta in the Far West cluster. There is reason to believe that such analyses could capture additional clusters as salicologists have previously described shrub willow west of the Rocky Mountains and in the northwestern boreal region as a separate species, *S. prolixa* (previously *S. eriocephala* ssp. *mackenzieana*)^12^*.* Moreover, in this scenario, the Alberta genotypes in the Far West cluster could perhaps represent a transition between *S. prolixa* and *S. famelica*.

After the last glacial retreat, range expansion and recolonization patterns may have contributed to the modern-day regional population structure in *S. famelica* and *S. eriocephala*. In addition, the barrier of the Great Lakes and the Canadian Shield along the longitudinal axis could plausibly limit gene flow between western and eastern populations. However, more genotypes from areas north of the Great Lakes throughout Ontario and southern Quebec are needed to validate this hypothesis. Evident genetic discontinuities along latitudinal and longitudinal transects have previously been observed for other North American tree and shrub species, including *Pinus contorta*, *Salix melanopsis, Populus trichocarpa, and Populus balsamifera*^57–60^.

With the evidence at hand, it appears that germplasm collected from Ontario may represent a zone of transition between western (*S. famelica*) and eastern (*S. eriocephala*) genotypes. Of particular note are the genotypes from Batchawana Bay in Ontario in which significant admixture was observed with genotypes from the West, Central, East, and even Far East clusters. The genotypes from Newfoundland and Labrador are also noteworthy as they appear to be the most divergent, an observation which conforms with decades of research on the genetic isolation of Newfoundland flora^61^.

### Genetic variation within the AgCan*Salix* collection

High recombination rates among outcrossing and sexually propagated plant species generally result in high genetic diversity. Among the genotypes sampled in this study, the observed heterozygosity (*H*_*O*_) ranged from 0.125 to 0.162 for the five clusters that were identified by Bayesian analysis. Comparable levels of *H*_*O*_ were reported among 45 genotypes of *S. eriocephala* collected from Ontario (mean *H*_*O*_ = 0.136;^62^). Conversely, 58 genotypes collected from Quebec and New Brunswick exhibited higher heterozygosity (mean *H*_*O*_ = 0.592;^63^). However, it is difficult to directly compare SNP-based studies on genetic diversity, such as ours, with earlier techniques.

Generally, levels of genetic differentiation are relatively lower among wind-pollinated taxa than for insect-pollinated species. Shrub willow are mainly, but not exclusively, insect-pollinated^64^. Within the AgCan*Salix* collection, we observed an overall *F*_*ST*_ = 0.024. An earlier microsatellites analysis by Lin et al*.*^65^ reported a slightly higher degree of differentiation (*F*_*ST*_ = 0.055) for 416 genotypes of *S. eriocephala* collected from three watersheds in upstate New York, USA. *S. silicicola*, a willow species endemic to the sand dunes of northern Saskatchewan, also displayed a higher *F*_*ST*_ = 0.156 in a study of 204 genotypes^66^.

The Far East cluster showed the highest levels of heterozygosity and also had the lowest *F*_*ST*_ values in comparison with all other clusters. This observation challenges the results of the population structure analyses which suggested that this cluster is the most divergent. It could be that the smaller sample size in this cluster is the source of the high level of heterozygosity. These contrasting observations bring into question the validity of the Far East cluster, particularly given the vast geographic distances between many of these genotypes. Of the 30 genotypes from Newfoundland, only five (from Stephenville) grouped in the Far East cluster, along with two genotypes from Ontario (Batchawana Bay and Kenora), and one from Quebec (Quebec City). Further analyses of the Far East genotypes and of additional germplasm from Newfoundland are needed to assess the extent of genetic differentiation in this region.

Analysis of isolation-by-distance revealed that genetic diversity was strongly correlated with the geographic distance between genotypes. However, the AMOVA revealed that the greatest variance occurred within genotypes (68.9%), while variance among the five clusters represented only 8.2%, indicating that *S. famelica* and *S. eriocephala* are highly outcrossing species. Previous molecular genetics studies on *S. eriocephala* have also reported that, although there exists a high level of allelic diversity, the majority of genetic variation occurs within populations and subpopulations rather than between populations^62,65^. Keller et al.^60^ analysed a collection of *Populus balsamifera*, a species with a comparably extensive range, and similarly found a low variance (4.4%) among groups. Such patterns of genetic diversity offer both challenges and opportunities for breeding efforts since highly heterozygous outcrossing populations can exhibit diverse phenotypes in crossings, but it can be difficult to tease apart adaptive alleles of interest from the abundant neutral genetic diversity.

### Biogeography of *Salix famelica* and *Salix eriocephala*

A redundancy analysis that asked whether biogeographic factors can help explain some of the genetic diversity and population structure pointed to precipitation, frost tolerance, and summer temperatures. This is hardly surprising given the vastly different climates that occur across the west–east spatial separation of the clusters. A single willow plant produces thousands of small seeds which lack storage reserves and require suitably mesic sites for establishment. Since both *S. famelica* and *S. eriocephala* rely on wind for long-distance pollen and seed dispersal^67^, extensive gene flow could theoretically occur between distant populations. More locally, shrub willow are prone to extensive clonal reproduction since broken twigs and branches frequently root and take hold nearby^68^.

While western sites of origin in this study receive less annual precipitation compared to eastern sites, they are also exposed to more extreme summer and winter temperatures. Higher precipitation coupled with high soil moisture offer biologically suitable habitats for seed germination and seedling establishment. On average, the sites of origin from the Central, East, and Far East clusters received two-and-a-half times more precipitation annually (1,171 mm) compared to West and Far West sites of origin (468 mm). Labrecque and Teodorescu^69^ found that the growth of shrub willow in southern Quebec was evidently more limited by precipitation than by summer temperatures. On the other hand, western genotypes which occur in more continental climes may be better adapted to hotter, drier conditions in spring and summer and could be more resistant to frost damage in winter. Western sites of origin had MST values ranging from 11.2 to 15.5 °C and eastern sites ranged from 9.4 to 15.4 °C, while the average number of FFD was 189 for the eastern sites of origin compared to 166 for western sites. It is highly plausible that the west–east differences in climate contributed to the divergence of *S. famelica* and *S. eriocephala*. Of course, the willow genotypes examined in this study occur across gradients of climate and geography such that genotypes in the Central cluster may be adapted to intermediate conditions between the western and eastern regions.

Although geoclimatic variables clearly influenced the diversification of Canadian shrub willow, the enduring question is whether genetics trump environmental conditions with regards to performance in SRC and environmental applications. While the growth and yield of willow clones showed strong genetic-by-environmental interactions in common garden coppicing trials, *S. eriocephala* genotypes consistently performed the best on more productive sites^70^. However, in a study on the effectiveness of willow in phytoremediation of contaminated soils from industrial sites, it was observed that genetics accounted for the majority of performance differences^71^. Accordingly, it will be vital to account for the effects of local growing conditions when testing the performance of novel cultivars by conducting breeding trials in common garden experiments.

## Conclusions

Opportunities abound to breed advanced willow cultivars by exploiting the expansive genetic diversity that exists across the ranges of *S. famelica* and *S. eriocephala*. One of the goals in establishing the AgCan*Salix* collection was to identify traits that influence climate adaptation, particularly with regards to drought, frost, and thermal stresses. Given that these are evidently key drivers for diversity in *S. famelica* and *S. eriocephala*, it should be eminently feasible to select for such traits in controlled crosses. Ultimately, the aim is to develop cultivars that are better suited to warmer and drier environments, particularly in the face of rapid global climate change.

In many ways, the use of Canadian shrub willow in SRC and environmental applications is still in its infancy. *S. eriocephala* has been promoted for bioenergy production as it is known to produce good yields of high-quality biomass^4,72^. Future breeding work with the AgCan*Salix* collection will seek to prioritize traits for high productivity in SRC. There have already been some successes in identifying genotypes of *S. eriocephala* for greater biomass yields^73^ and for phytoremediation applications as well^74^. However, the breeding work to date has been limited to a relatively small number of genotypes, just a fraction of those available in the AgCan*Salix* collection.

This study helps lay the groundwork for future controlled crossing experiments. Locally adapted genotypes that are more resilient to environmental and biotic stresses could be selected by exploiting the allelic diversity of the AgCan*Salix* collection. For example, genotypes with improved resistance to insect herbivores were readily identified among a collection of *S. eriocephala* hybrids^75^. However, the challenge of selecting and maintaining such adaptive traits from highly heterozygous outcrossing populations must not be underestimated^76^*.* The next steps will be to identify candidate genotypes for breeding in order to harness the inherent diversity of Canadian shrub willow and advance efforts to develop elite cultivars.

## Supplementary Information

Supplementary Information.

## Data Availability

The datasets generated during and/or analysed during the current study are available in the Dryad repository, https://doi.org/10.5061/dryad.905qfttp7.
