# Supplementary material for: Unweaving the population structure and genetic diversity of Canadian shrub willow
Source: Sci Rep. 2022 Oct 14;12:17254. doi: 10.1038/s41598-022-20498-9 (PMC9568530; doi:10.1038/s41598-022-20498-9)
Supplement: Supplementary file 1 — Supplementary Information. [file 41598_2022_20498_MOESM1_ESM.docx]

# SUPPLEMENTARY INFORMATION

# Unweaving the population structure and genetic diversity of Canadian shrub willow

# Emily K. Murphy, Eduardo P. Cappa, Raju Y. Soolanayakanahally, Yousry A. El-Kassaby, Isobel A. P. Parkin, William R. Schroeder, and Shawn D. Mansfield

**Table S1.** Location and climatic data for 33 sites of origin for the *S. famelica* and *S. eriocephala* genotypes in the AgCan*Salix* collection*.* The sites of origin are arranged from west to east. In this study, 8 – 10 genotypes were collected at each site of origin for a total of 324 genotypes.

| **Code** | **Site of Origin** | **Province^†^** | **LAT^‡^** | **LON^‡^** | **ELEV^‡^** | **MAP^‡^** | **MST^‡^** | **FFD^‡^** | **GDD^‡^** |
| --- | --- | --- | --- | --- | --- | --- | --- | --- | --- |
|  |  |  | **(°N)** | **(°W)** | **(m)** | **(mm)** | **(°C)** | **(days)** | **(°C)** |
| TAB | Taber | AB | 49.48 | 112.1 | 754 | 316 | 14.9 | 170 | 1717 |
| BOW | Bow River | AB | 50.47 | 112.38 | 791 | 515 | 13.6 | 169 | 1490 |
| DRU | Drumheller | AB | 51.28 | 112.43 | 683 | 338 | 13.9 | 160 | 1533 |
| STL | Stettler | AB | 52.17 | 113.15 | 800 | 478 | 13.6 | 159 | 1446 |
| CAM | Camrose | AB | 52.55 | 112.51 | 689 | 438 | 13.6 | 165 | 1431 |
| KIP | Kipling | SK | 49.55 | 102.37 | 751 | 396 | 15.5 | 175 | 1848 |
| MJW | Moose Jaw | SK | 50.22 | 105.49 | 669 | 365.1 | 14.1 | 176 | 1845 |
| IHD | Indian Head | SK | 50.30 | 103.40 | 602 | 447.3 | 13.3 | 164 | 1671 |
| WAT | Watrous | SK | 51.35 | 105.27 | 550 | 434.5 | 13.0 | 163 | 1628 |
| WAK | Wakaw | SK | 52.41 | 105.43 | 528 | 388 | 12.3 | 161 | 1517 |
| CLK | Christopher Lake | SK | 53.30 | 105.45 | 531 | 424.3 | 12.1 | 154 | 1467 |
| LAR | La Ronge | SK | 55.00 | 105.21 | 390 | 483.8 | 11.2 | 151 | 1323 |
| MDN | Morden | MB | 49.00 | 98.08 | 431 | 533.3 | 14.6 | 183 | 1955 |
| POR | Portage La Prairie | MB | 49.59 | 98.16 | 256 | 514.5 | 13.6 | 167 | 1784 |
| ASH | Ashern | MB | 51.07 | 98.22 | 264 | 499.9 | 12.6 | 159 | 1588 |
| SMJ | St. Martin Junction | MB | 51.43 | 98.50 | 251 | 499.9 | 12.6 | 159 | 1588 |
| EAS | Easterville | MB | 52.55 | 99.10 | 282 | 442.8 | 11.5 | 158 | 1407 |
| GRR | Grand Rapids | MB | 53.51 | 99.14 | 280 | 473.7 | 13.0 | 167 | 1508 |
| STU | Sturgeon River | ON | 46.31 | 80.01 | 231 | 899.3 | 13.3 | 183 | 1705 |
| BAT | Batchawana Bay | ON | 46.54 | 84.35 | 169 | 1011 | 13.3 | 195 | 1718 |
| TBY | Thunder Bay | ON | 48.31 | 88.43 | 231 | 711.6 | 12.0 | 162 | 1434 |
| FTF | Fort Frances | ON | 48.38 | 93.19 | 345 | 709.5 | 14.0 | 180 | 1821 |
| FAQ | Fauquier | ON | 49.18 | 82.02 | 224 | 831.8 | 11.2 | 151 | 1370 |
| KEN | Kenora | ON | 49.48 | 94.30 | 344 | 447.3 | 13.3 | 164 | 1671 |
| QUE | Quebec | QC | 46.98 | 70.71 | 15 | 1141 | 15.4 | 184 | 1707 |
| NBS | NB South | NB | 45.32 | 66.03 | 8 | 1154 | 14.2 | 210 | 1891 |
| NBC | NB Central | NB | 46.13 | 67.42 | 116 | 1114 | 13.7 | 178 | 1768 |
| NBN | NB North | NB | 47.11 | 67.56 | 153 | 1134 | 12.6 | 181 | 1577 |
| NSW | NS West | NS | 45.21 | 63.16 | 21 | 1202 | 13.0 | 193 | 1684 |
| NSE | NS East | NS | 45.52 | 61.15 | 24 | 1505 | 12.0 | 197 | 1541 |
| PEI | Prince Edward Island | PEI | 46.26 | 62.01 | 21 | 1078 | 13.1 | 204 | 1722 |
| COD | Codroy Valley | NL | 47.51 | 59.18 | 4 | 1709 | 9.4 | 200 | 1023 |
| STE | Stephenville | NL | 48.33 | 58.40 | 8 | 1352 | 11.0 | 204 | 1324 |
| DER | Deer Lake | NL | 49.10 | 57.37 | 12 | 1127 | 10.9 | 180 | 1298 |

^†^AB, Alberta; SK, Saskatchewan; MB, Manitoba; ON, Ontario; QC, Quebec; NB, New Brunswick; NS, Nova Scotia; PEI, Prince Edward Island; NL, Newfoundland and Labrador.

^‡^LAT, latitude; LON, longitude; ELEV, elevation; MAP, mean annual precipitation; MST, mean summer temperature; FFD, frost-free days; and GDD, degree days above 5**°**C.
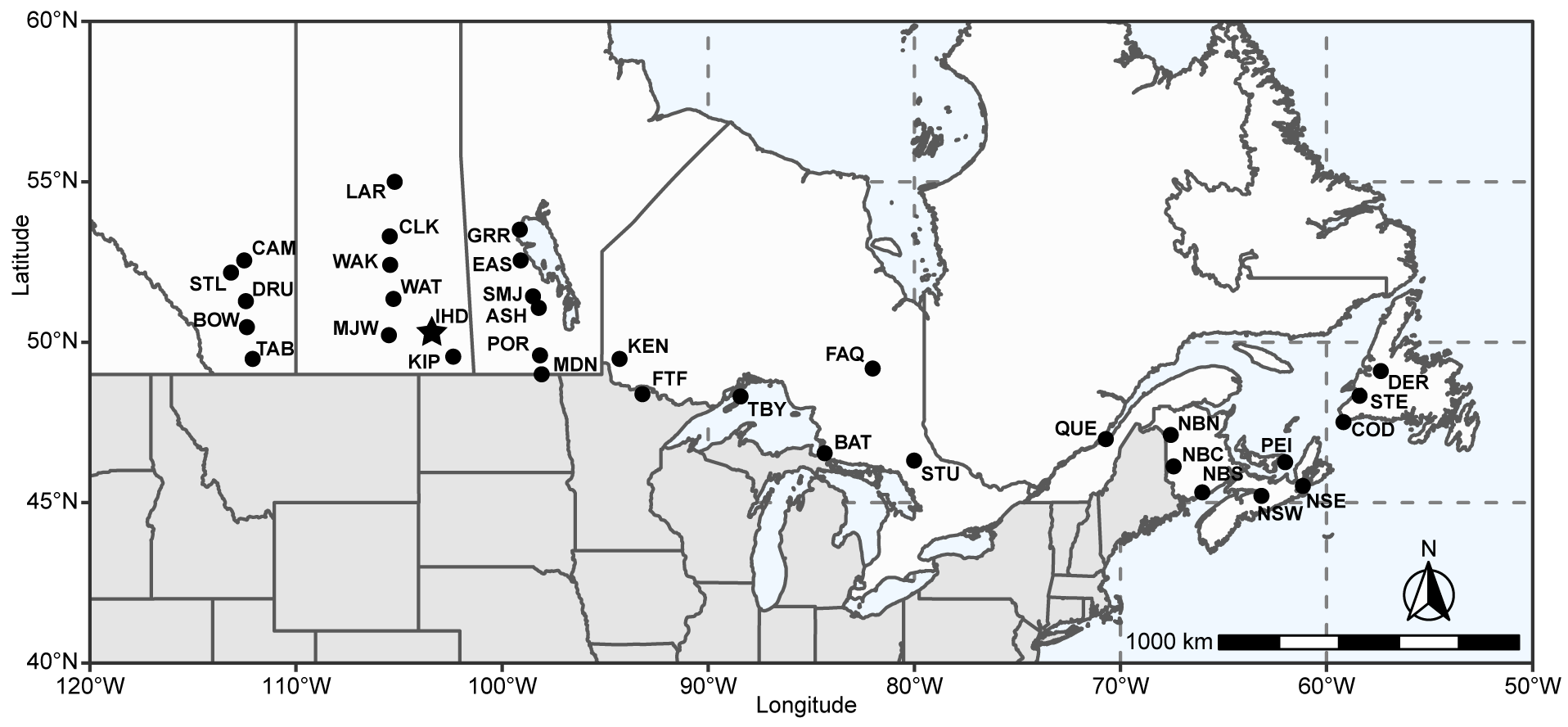


**Supplemental Figure S1.** Distribution of 33 sites of origin for 324 *S. famelica* and *S. eriocephala* genotypes employed in this study. The common garden plot was established at Indian Head, Saskatchewan (black star).

**
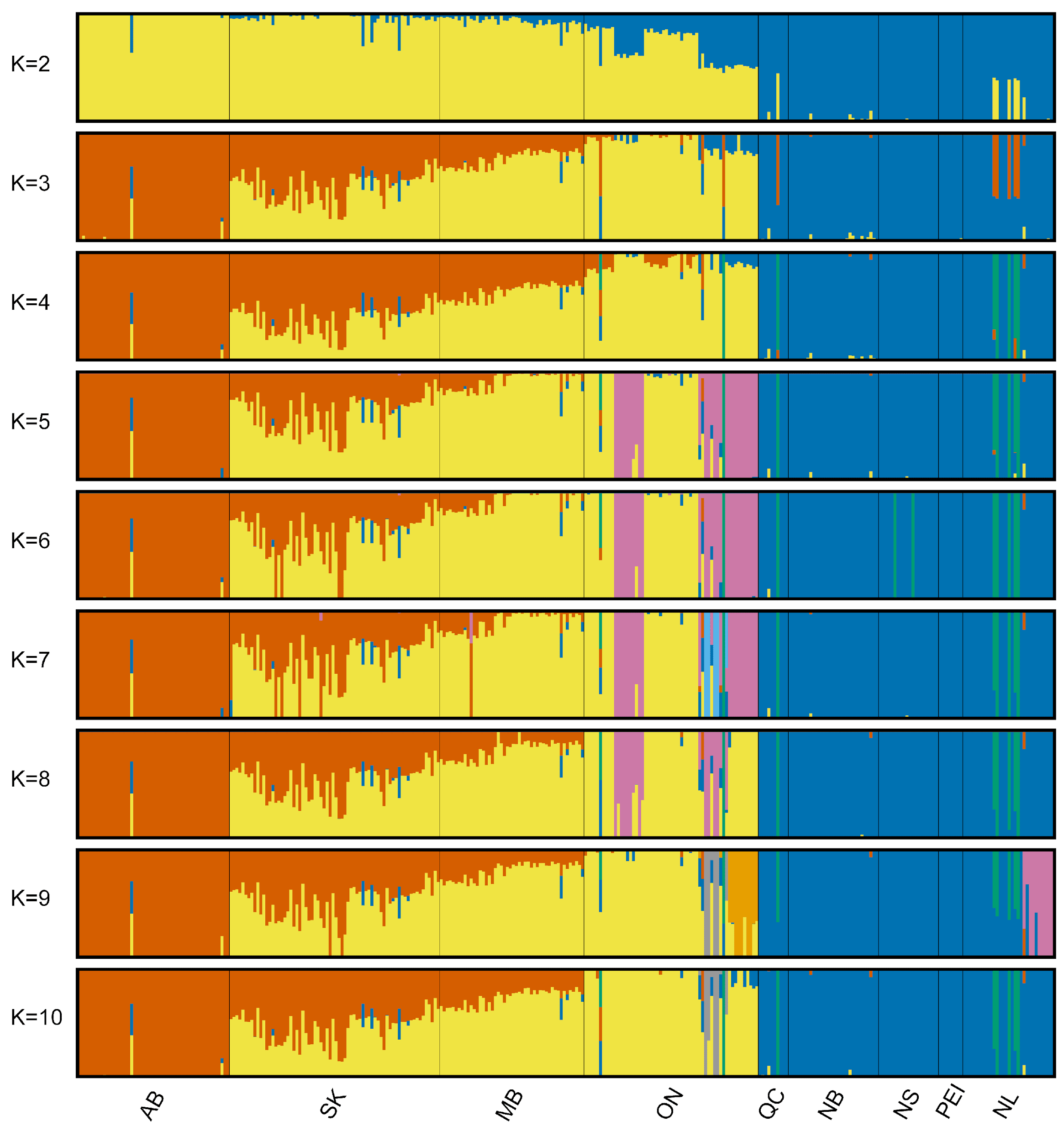
**

**Supplementary Figure S2.** Distribution of fastSTRUCTURE-defined clusters among 324 genotypes, sorted from west to east, for *K* values 2 – 10 where the y-axis indicates the estimated admixture coefficients for each genotype.

**
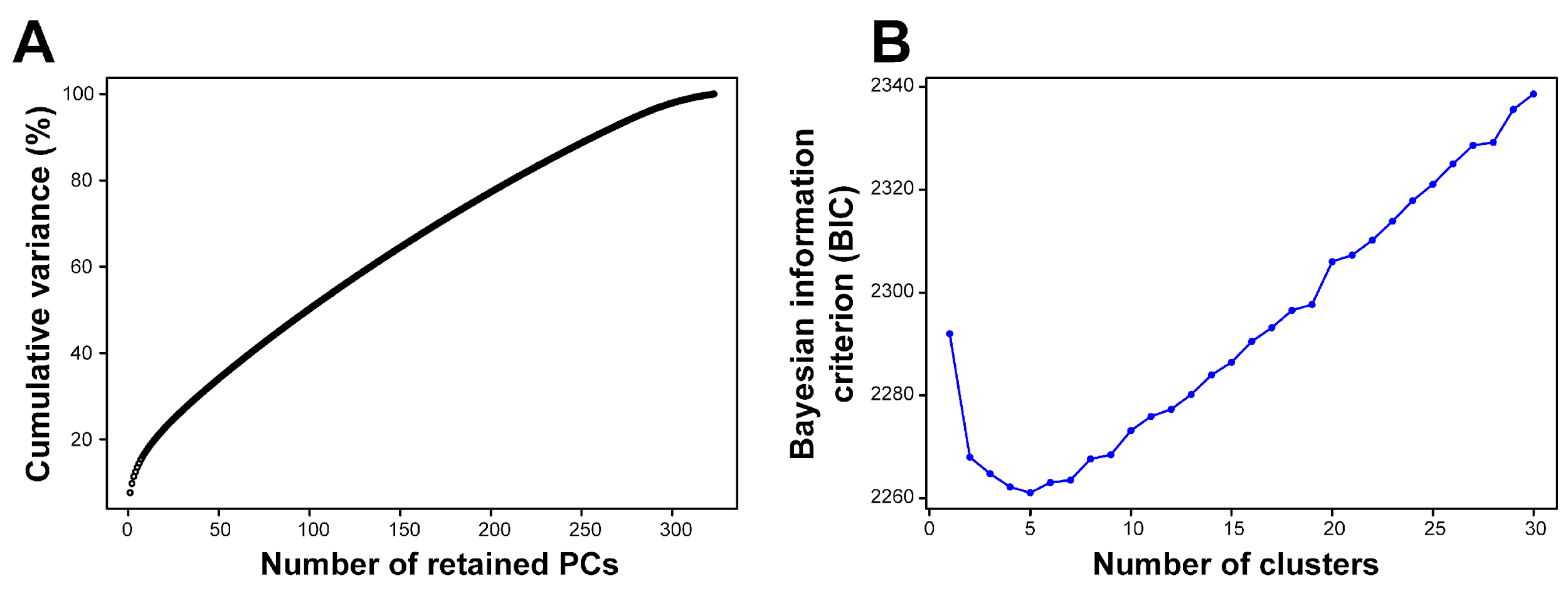
**

**Supplementary Figure S3.** Inference of the optimal number of clusters (*K*) for 324 accessions from 33 populations using the *find.clusters* function in the R package *adegenet*. A) The cumulative percentage of the total variance for the number of retained principal components (PCs). B) The Bayesian information criterion (BIC) for successive numbers of clusters (from *K* = 1 to *K* = 30) for all individuals without prior clustering.


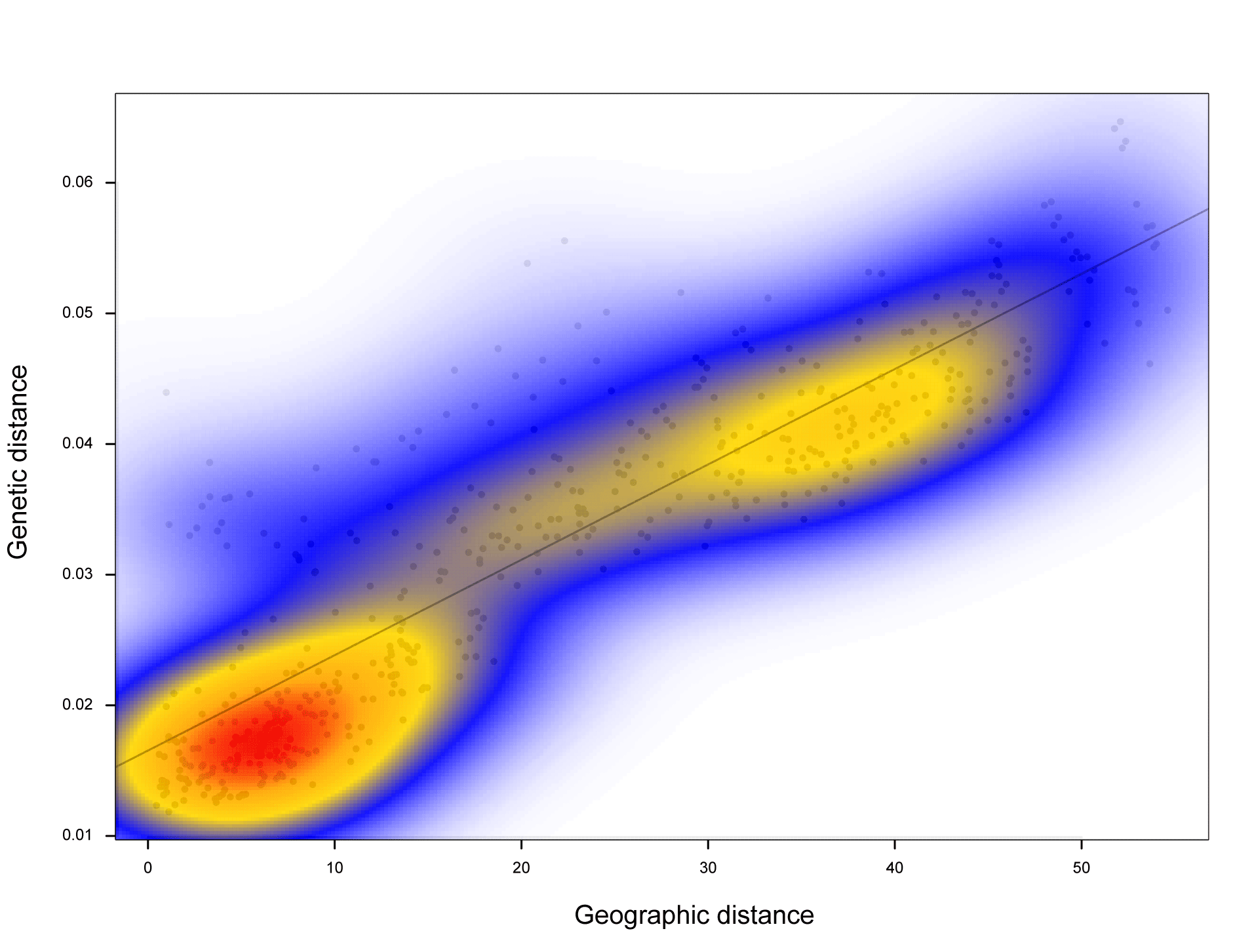


**Supplementary Figure S4.** Isolation by distance plot showing the pairwise Edwards genetic distance between genotypes plotted against Euclidean geographical distances for 324 genotypes of *S. famelica* and *S. eriocephala* (*r* = 0.887, *p* = 0.001). Local density is illustrated using the two-dimensional kernel density estimation in R package *MASS* where red represents high density and blue represents low density.
